# Pharmacological management in frontotemporal dementia and patient outcomes: a retrospective cohort study using the TriNetX platform

**DOI:** 10.3389/fneur.2026.1930046

**Published:** 2026-09-03

**Authors:** Lorenza Pabst Botton, Fernando Jacob Lazzaretti, Carlos Lokschin, Tharick A. Pascoal, Jaderson Costa da Costa, Bruce L. Miller, Lucas P. Schilling, Cristiano S. Aguzzoli

**Affiliations:** 1 School of Medicine, Pontifical Catholic University of Rio Grande do Sul (PUCRS), Porto Alegre, Brazil; 2 Department of Psychiatry, University of Pittsburgh, Pittsburgh, PA, United States; 3 Department of Neurology, São Lucas Hospital of PUCRS, Porto Alegre, Brazil; 4 Brain Institute of Rio Grande do Sul (BraIns), Porto Alegre, Brazil; 5 Global Brain Health Institute, Memory and Aging Center, University of California, San Francisco, San Francisco, CA, United States; 6 Department of Neurology, University of California, San Francisco, San Francisco, CA, United States

**Keywords:** clinical outcomes, frontotemporal dementia, hospital-based acute care, neuropsychiatric medications, pharmacological symptom management

## Abstract

Symptomatic treatment remains the cornerstone of therapeutic management in frontotemporal dementia (FTD), as disease-modifying therapies are currently unavailable. However, several medications originally developed for other neuropsychiatric conditions may represent potentially harmful medications (PHMs) in FTD. This study aimed to describe prescribing patterns following an FTD diagnosis and to evaluate the association between PHM exposure and hospital-based acute care (HBAC) utilization. We conducted a retrospective cohort study using TriNetX, a global health research network comprising clinical data from real-world settings. We included 179 individuals with an electronic health record (EHR)-recorded diagnosis of FTD from 43 healthcare organizations across the United States. Propensity-score matching balanced baseline characteristics, with propensity scores estimated by logistic regression. Risk ratios were calculated from observed event proportions in the matched cohorts, and time to first event was compared using Kaplan–Meier estimates and a Cox proportional hazards model. Patients with at least one recorded PHM prescription after diagnosis formed the exposed cohort; those with none formed the unexposed cohort. The two cohorts were matched 1:1 using propensity scores to minimize confounding and to balance key demographic and clinical variables between groups. HBAC was defined as hospital inpatient or observation care, emergency department visits, or critical care services. Among the 179 individuals with FTD (mean age 67.9 ± 14.9 years; 48.0% women), 71 (39.7%) were exposed to PHMs. After propensity-score matching, baseline characteristics were well balanced (56 patients per group). Overall, 39.7% of patients received at least one PHM, most commonly benzodiazepines (69.5%). In matched analyses, PHM exposure was associated with a significantly higher risk of HBAC [risk ratio (RR) = 2.20; 95% confidence interval (CI), 1.35–3.57] and lower event-free survival (12.8% vs. 62.7%). This real-world study of post-diagnosis prescribing in FTD shows that PHM use is common and that a greater proportion of exposed patients experienced HBAC, with a shorter time to the first event; given residual confounding by indication, these findings are exploratory and hypothesis-generating rather than causal.

## Introduction

Frontotemporal dementia (FTD) is a heterogeneous neurodegenerative syndrome marked by frontal and temporal atrophy that affects behavior and language, with three variants: behavioral variant FTD (bvFTD), semantic variant primary progressive aphasia (PPA), and non-fluent variant PPA (1). The behavioral variant is the most common phenotypic presentation, accounting for approximately 50% of FTD cases, and is associated with apathy, loss of empathy, compulsive behavior, disinhibition, appetite disturbances, and executive dysfunction (2, 3).

Without disease-modifying therapy, symptomatic treatment is the mainstay and aims to improve the quality of life for patients and caregivers. Treatment decision-making in FTD is based on the clinical syndrome and the stage of the disease. Current evidence suggests that drugs such as selective serotonin reuptake inhibitors (SSRIs) appear to promote symptom relief in FTD; the strongest behavioral evidence, from a small randomized trial, supports trazodone (4). Yet, in the absence of standardized treatment protocols, prescribing practices targeting symptoms are largely empirical and often include potentially harmful medications (PHMs), such as antipsychotics, benzodiazepines, and Alzheimer’s disease medications (cholinesterase inhibitors and memantine) (5–8). Evidence supporting these drug classes in FTD is limited, and prior studies have raised concerns regarding their tolerability and adverse effects in dementia populations, as these medications may aggravate core behavioral symptoms such as agitation, disinhibition, impulsivity, apathy, and confusion (4, 7, 9–15). However, whether PHM exposure also increases the risk of hospital-based acute care (HBAC) in FTD is unknown. In this study, we aimed to describe prescribing patterns following an FTD diagnosis and to evaluate whether PHM exposure is associated with increased HBAC utilization.

## Methods

This study follows the Strengthening the Reporting of Observational Studies in Epidemiology (STROBE) (16) reporting guideline for cohort studies.

### Data source

We used the TriNetX Research Network (accessed 21 July 2025), a federated network of de-identified electronic health record (EHR) data from 107 healthcare organizations across the United States. Individuals from 43 organizations across the United States met the eligibility criteria for this study. As data in the network are de-identified at the source, informed consent was waived. Analyses were conducted within TriNetX using the Lines of Treatment and Compare Outcomes modules under identical eligibility filters. Further information on the TriNetX protocol used is presented in the Supplementary material, Section E1.

### Study design and population

We conducted a retrospective cohort study of adults (≥18 years) with a diagnosis of FTD based on the corresponding International Classification of Diseases, 10th Revision (ICD-10) code (FTD; ICD-10 G31.0) recorded during an outpatient visit within the 5 years preceding the analysis date. Patients were required to have structural brain imaging with computed tomography (CT) or magnetic resonance imaging (MRI) on or after the diagnosis date. We excluded individuals with other neurodegenerative conditions and those with unspecified dementia ICD codes (3). The index date was the first qualifying EHR-recorded diagnosis of FTD. For the exposed cohort in Compare Outcomes, because a qualifying prescription within 1 year of diagnosis was itself part of the cohort-defining criteria, the index event reflects the FTD diagnosis in conjunction with this treatment criterion (see Statistical Analysis and Limitations sections). In addition, because of platform limitations, the specific FTD variant for each patient could not be specified, and the network does not distinguish behavioral-variant FTD (bvFTD) from the primary progressive aphasia (PPA) variants.

**Figure 1:**
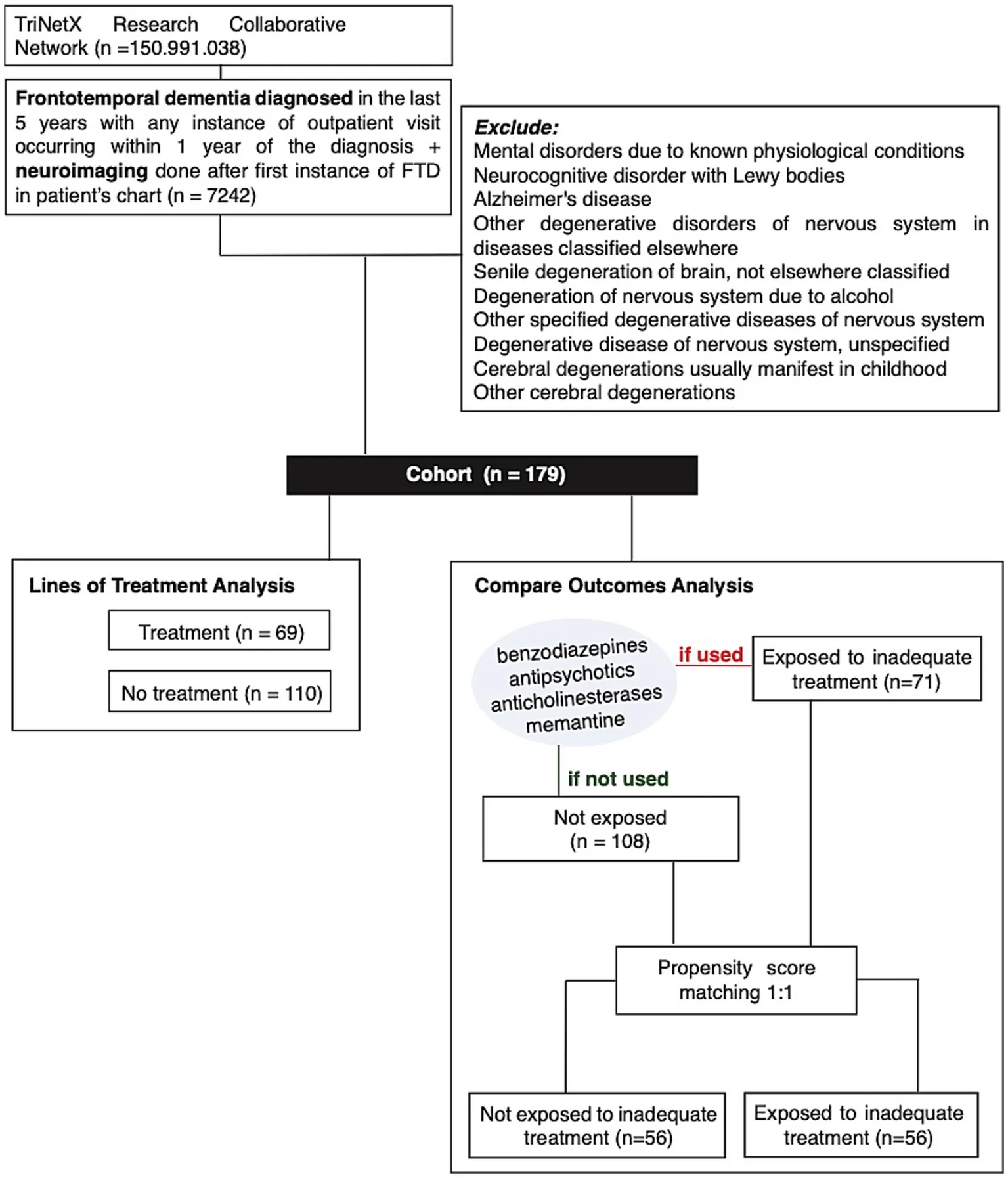
Study flowchart. The flowchart presents the study methodology. Criteria were applied sequentially; each box reports the number of patients removed at that step and the number remaining. Patients meeting more than one criterion are counted once, at the first criterion applied. FTD, frontotemporal dementia.

It is important to state that TriNetX applies exclusion criteria sequentially; therefore, a patient meeting more than one criterion is counted under the first criterion applied. Of the 7,242 patients who met the base criteria, 7,063 (97.5%) were excluded and 179 were retained. One criterion accounted for 99% of that attrition: 6,989 patients carried a code from the F01–F09 block (mental disorders due to known physiological conditions), which includes unspecified dementia (F03), delirium (F05), and dementia in diseases classified elsewhere (F02). The remaining criteria accounted for the exclusion of 74 patients. Figure 1 reports the count at each step and the patient selection flow; diagnostic ICD codes and detailed inclusion criteria are outlined in Supplementary Table 1.

### Exposures

The primary exposure was defined as the use of PHMs in patients with FTD within 1 year after the index date. PHMs included in the query were antipsychotics—quetiapine, risperidone, clozapine, olanzapine, aripiprazole, haloperidol, or chlorpromazine; benzodiazepines—diazepam, clonazepam, alprazolam, midazolam, or lorazepam; and Alzheimer’s disease drugs—donepezil, rivastigmine, galantamine, or memantine. We used the term PHMs for drug classes with limited efficacy evidence in FTD and documented safety concerns in dementia. We preferred this term to potentially inappropriate medications (PIMs), which, in the geriatric literature, denotes drugs identified by a validated explicit instrument such as the Beers Criteria, the Screening Tool of Older Persons’ Prescriptions (STOPP), or the EU(7)-PIM list. We applied no such instrument; our list was derived from systematic reviews of pharmacological management in FTD (8). The two constructs overlap without being equivalent.

Patients with ≥1 prescription from any of these classes formed the exposed cohort; those with none formed the unexposed cohort. All listed medications included in this study were selected based on prior systematic reviews and demographic studies related to symptom management in FTD. Importantly, trazodone, SSRIs (e.g., paroxetine, citalopram, and fluvoxamine), and oxytocin were reported in this descriptive summary but were not considered potentially harmful exposures. Using the Lines of Treatment module in TriNetX, we identified each patient’s treatment regimen initiated after diagnosis and defined them as either the “Treatment” or the “No treatment” group, with “Treatment” defined by the use of any of the medication classes included in the query.

### Outcomes and follow-up

The primary outcome was defined as HBAC utilization, a composite of hospital inpatient and observation care services, emergency department services, or critical care services (17). HBAC was assessed from day 1 after the index date until the first event or last recorded encounter, with follow-up capped at 10 years to standardize the maximum follow-up window. If the outcome did not occur during the analysis period, patients were censored at the time of their last recorded encounter. Using Compare Outcomes, we compared the risk of HBAC between the exposed and unexposed groups.

### Statistical analysis

**Figure 2:**
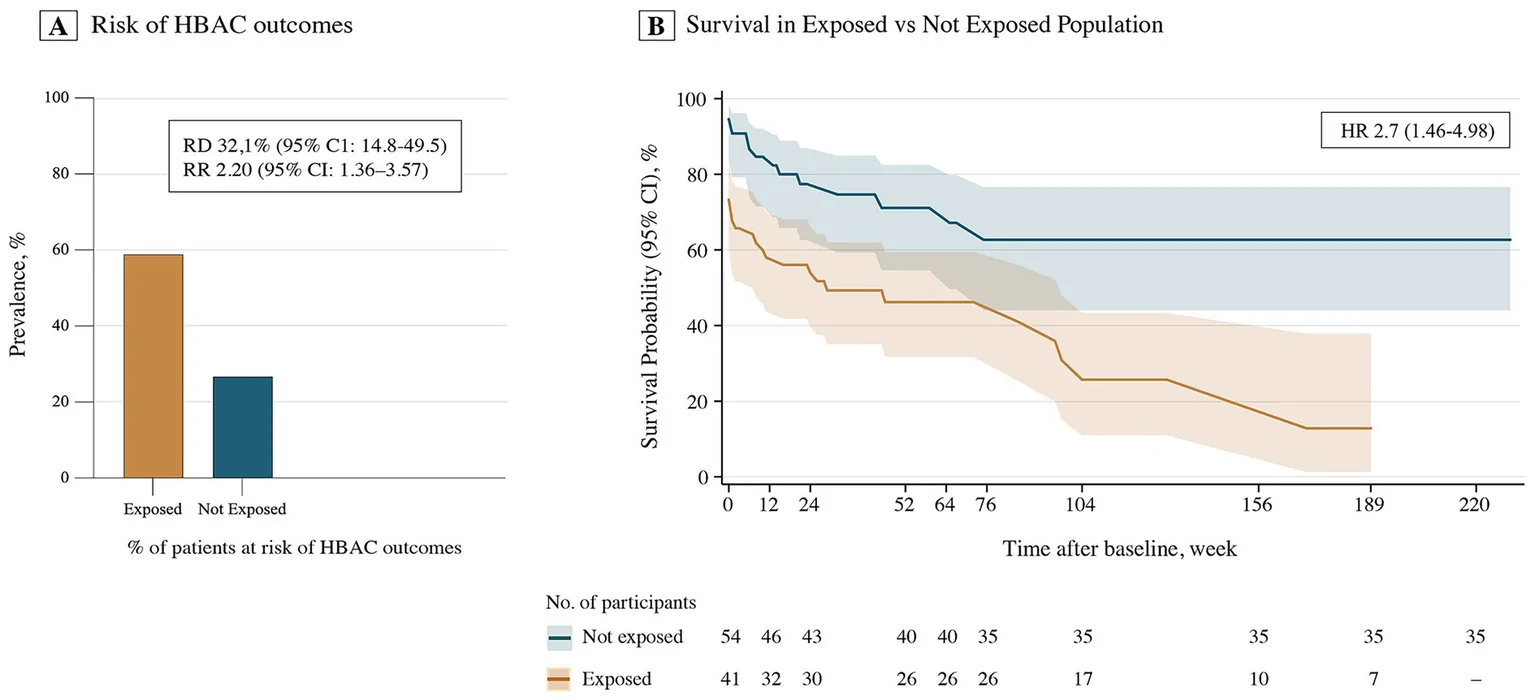
Risk of outcomes and survival analysis. After matching for age, sex, and comorbidities, individuals exposed to PHMs had an elevated risk of composite adverse outcomes (hospitalization, emergency department visits, or critical care) compared with those who were unexposed (RR = 2.20; 95% CI, 1.36– 3.57, panel **A**). Further survival analysis comparing the exposed and unexposed groups revealed a significant difference in survival (panel **B**). The exposed group had a significantly lower event-free probability (12.83% vs. 62.65%), with a median time to event of 201 days. The unexposed group did not reach the median time to event, indicating that more than 50% of patients remained event-free. The log-rank test was significant (p = 0.0009), indicating a 2.69-fold higher hazard in the exposed group. The number of participants at week 0 reflects patients without a qualifying event at or before their index date; patients with an earlier event remain in the analysis but are counted as having experienced the outcome at baseline. Values at later time points are calculated from cohort size × reported survival probability (see Statistical Analysis section). Estimates in the tail of the curve rest on few patients still at risk and are imprecise. CI, confidence interval; HR, hazard ratio; PHM, potentially harmful medication; RR, risk ratio.

We performed propensity-score matching to balance baseline covariates. Exposed and unexposed groups were matched on 17 index-date variables: current age, age at index, sex, race (White, Asian, and Black/African American), ethnicity (Hispanic/Latino, not Hispanic/Latino, or unknown), body mass index, and comorbidities (including factors influencing health-services contact; circulatory; endocrine/metabolic; neoplasms; mental/behavioral/neurodevelopmental; and external causes). Comorbidities were based on their respective ICD-10 codes. We reported counts (%) for categorical variables and means ± standard deviations (SD) for continuous variables and compared groups using χ^2^ or unpaired t-tests. We estimated the time to first HBAC event using a Cox proportional hazards model; patients were censored at their last recorded encounter. The Kaplan–Meier analysis included patients who had an outcome recorded before their index date rather than excluding them; such patients remained in the analysis but were counted as having experienced the event at baseline. As the exposed cohort’s index date may be determined by the qualifying prescription rather than by the diagnosis date (see Limitations section), an event occurring between diagnosis and prescription is therefore recorded as occurring prior to index for that patient. Participant counts at later time points in Figure 2 are calculated as cohort size × the survival probability reported by TriNetX at each interval, since the platform does not provide patient-level censoring data needed to construct these values from direct counts. Propensity scores were estimated by logistic regression on the 17 pre-index covariates; cohorts were matched 1:1 by greedy nearest-neighbor matching in TriNetX, with balance judged by standardized mean differences below 0.10. After propensity-score matching, measures of association, including risk ratios (RRs), odds ratios (ORs), risk differences, and 95% confidence intervals (CIs), were calculated using the TriNetX Compare Outcomes module based on the observed event risks in the matched cohorts. The TriNetX analytical framework applies a data privacy constraint that limits granularity: Any patient counts greater than zero but fewer than 10 are automatically rounded up to 10 to preserve anonymity. Nevertheless, survival analyses were conducted on sufficiently large cohorts (all *N* > 10), ensuring that this anonymity rule did not affect outcome estimates.

Lines of treatment and compare outcomes are computed independently in TriNetX. Both modules were configured to define exposure as ≥1 qualifying prescription within 1 year of diagnosis. Lines of Treatment identified 69 treated patients; the Compare Outcomes matched query returned 71 patients meeting the same exposure criteria. We were unable to fully reconcile this two-patient difference from the platform’s aggregate output alone, as TriNetX does not release patient-level records for direct comparison across modules. Descriptive prescribing patterns are reported from Lines of Treatment; the outcome comparison uses the Compare Outcomes cohorts. All proportions are reported with their denominator. Post-matching balance is shown in Table 1; additional details are provided in Supplementary Tables 1, 4, 5.

**Table 1:** Baseline demographic and clinical characteristics of study participants.

Characteristic	Before matching				After matching			
	Not exposed to PHM (*n* = 108)	Exposed to PHM (*n* = 71)	*p* value	Std. Diff.	Not exposed to PHM (*n* = 56)	Exposed to PHM (*n* = 56)	*p* value	Std. Diff.
Demographics								
Age (mean ± SD)	68.5 ± 14.8	67.0 ± 15.2	0.52	0.1	67.1 ± 15.4	66.0 ± 16.2	0.72	0.07
Age at index	66.1 ± 14.7	64.9 ± 15.3	0.59	0.08	64.9 ± 15.4	63.8 ± 16.2	0.72	0.07
Sex (%)								
Female	55 (50.9)	31 (43.7)	0.34	0.15	30 (53.6)	26 (46.4)	0.45	0.14
Ethnicity (%)								
White	84 (77.8)	45 (63.4)	0.04	0.32	38 (67.9)	40 (71.4)	0.68	0.08
Black or African American	10 (9.3)	12 (16.9)	0.13	0.23	10 (17.9)	10 (17.9)	1.00	<0.001
Asian	10 (9.3)	10 (14.1)	0.32	0.15	10 (17.9)	10 (17.9)	1.00	<0.001
Other ethnicities	10 (9.3)	10 (14.1)	0.32	0.15	10 (17.9)	10 (17.9)	1.00	<0.001
Unknown ethnicity	10 (9.3)	10 (14.1)	0.32	0.15	10 (17.9)	10 (17.9)	1.00	<0.001
Ethnicity (%)								
Hispanic or Latino	10 (9.3)	10 (14.1)	0.32	0.15	10 (17.9)	10 (17.9)	1.00	<0.001
Not Hispanic or Latino	83 (76.9)	59 (83.1)	0.31	0.16	46 (82.1)	46 (82.1)	1.00	<0.001
Unknown ethnicity	18 (16.7)	10 (14.1)	0.64	0.07	10 (17.9)	10 (17.9)	1.00	<0.001
Diagnosis (%)								
Factors influencing health status and contact with health services	71 (65.7)	49 (69.0)	0.65	0.07	38 (67.9)	37 (66.1)	0.84	0.04
Diseases of the circulatory system	58 (53.7)	51 (71.8)	0.01	0.38	35 (62.5)	36 (64.3)	0.84	0.04
Endocrine, nutritional, and metabolic diseases	69 (63.9)	54 (76.1)	0.09	0.27	41 (73.2)	40 (71.4)	0.83	0.04
Neoplasms	29 (26.9)	23 (32.4)	0.42	0.12	17 (30.4)	18 (32.1)	0.84	0.04
Mental, behavioral, and neurodevelopmental disorders	43 (39.8)	39 (54.9)	0.05	0.31	27 (48.2)	27 (48.2)	1.00	<0.001
External causes of morbidity	23 (21.3)	28 (39.4)	0.01	0.40	18 (32.1)	17 (30.4)	0.84	0.04
BMI (mean ± SD)	26.1 ± 6.1	27.7 ± 5.0	0.18	0.27	25.3 ± 6.5	27.2 ± 4.4	0.17	0.33
Procedure (%)								
Hospital Inpatient and Observation Care Services	32 (29.6)	29 (40.8)	0.12	0.24	21 (37.5)	21 (37.5)	1.00	<0.001
Emergency Department Services	43 (39.8)	35 (49.3)	0.21	0.19	27 (48.2)	24 (42.9)	0.57	0.11
Critical Care Services	10 (9.3)	16 (22.5)	0.01	0.37	10 (17.9)	13 (23.2)	0.48	0.13

Data are presented as counts and percentages for categorical variables or means ± standard deviations for continuous variables. Between-group differences were assessed using the chi-square tests for categorical variables and unpaired t-tests for continuous variables. Cells reported as 10 reflect the platform anonymity rule, under which counts between 1 and 9 are rounded up to 10. Standardized differences and p-values computed from those cells do not indicate exact balance. BMI, body mass index; PHM, potentially harmful medication; SD, standard deviation; Std. Diff., standardized difference.

### Ethics statement

This study used de-identified data from the TriNetX research network. As data in the TriNetX Research Network are de-identified at the source, informed consent and institutional review board approval were waived.

## Results

### Demographics

Of the 179 individuals evaluated before matching [mean (SD) age at baseline = 67.9 (14.9) years], 71 (39.7%) were exposed to PHMs, and 108 (60.3%) were not. Women represented 48.0% of the total sample (86/179), with similar proportions across exposure groups (43.7% exposed vs. 50.9% not exposed). A majority of the participants were white (129/179, 72.1%), followed by Black or African American (22/179, 12.3%), Asian (20/179, 11.2%), and Hispanic or Latino (20/179, 11.2%). Before the index date, 61 patients (34.1%) had an inpatient or observation encounter, 78 (43.6%) had an emergency department visit, and 26 (14.5%) had a critical care admission. These pre-index measures entered the propensity model as matching covariates. After matching, baseline characteristics were well balanced across exposure groups (*n* = 56 in each group), with no statistically significant differences in demographics, diagnoses, or healthcare utilization. Descriptive characteristics are shown in Table 1. Further demographic characteristics and propensity score-matched diagnoses are available in the Supplementary Table 8.

### Prescribing patterns after FTD diagnosis

**Figure 3:**
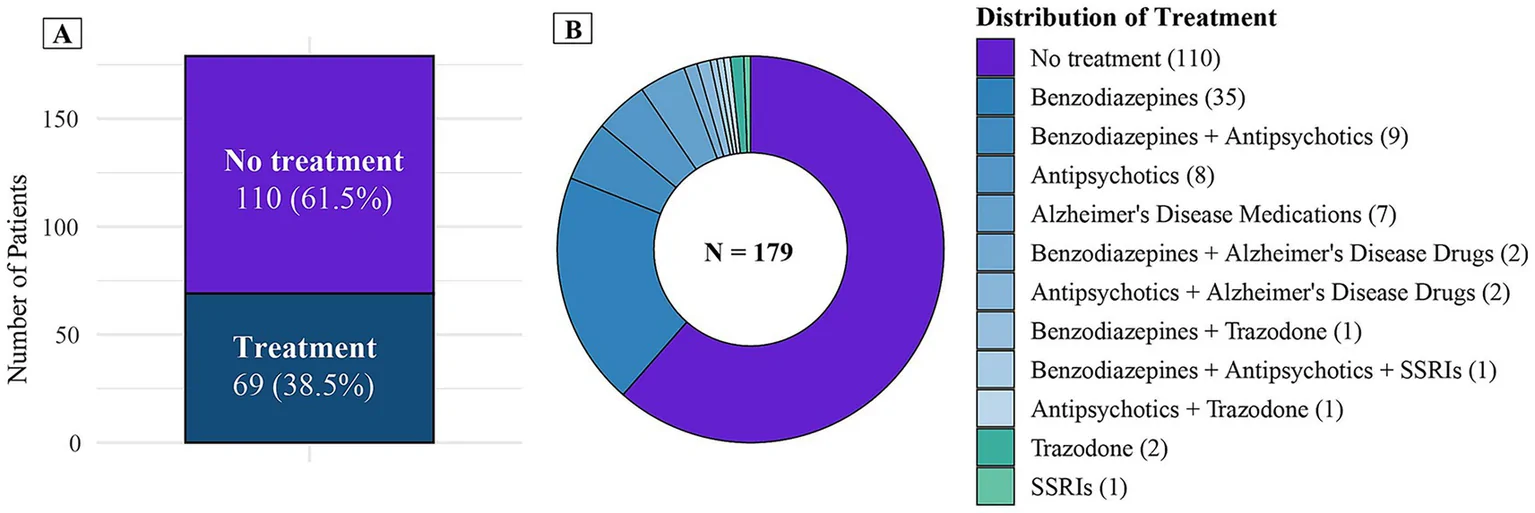
Lines of treatment. These graphs provide insight into the chosen pharmacological strategies used for frontotemporal dementia symptom management following diagnosis (total *N* = 179). Panel **(A)** shows the total number of patients who did and did not receive any medications included in the query. Panel **(B)** breaks down the subgroup of treated patients (*n* = 69) by pharmacological regimen. Patients who received no pharmacological treatment or medications not included in the LOT module are grouped as “No treatment.” LOT, lines of treatment; PHM, potentially harmful medication.

We found that 110 patients (61.5%) did not receive any qualifying pharmacological treatment included in the query (Figure 3A). The remaining 69 patients (38.5%) received at least one qualifying medication, as shown in Figure 3B. Benzodiazepines were the most frequently prescribed medication among the treatment group (69.5%), followed by antipsychotics (30.4%) and Alzheimer’s disease medications (15.9%). Only four patients were prescribed trazodone, and in two of these cases, it was used alongside PHMs. SSRIs were prescribed to two patients. The specific medications prescribed within each class are provided in Supplementary Table 3.

### Comparison of outcomes

This analysis included 71 patients in the exposed group and 108 in the unexposed group, based on the query criteria. When comparing the time to the composite outcome of hospital inpatient and observation care services, emergency department services, or critical care services, the mean follow-up time for the exposed group was 435 ± 394 days (median 322 days), whereas that for the unexposed group was 520 ± 481 days (median 374 days); see Supplementary Table 9.

### Exposure to PHM and risk of HBAC

After matching, individuals exposed to PHMs had an elevated risk of hospitalization, an emergency department visit, or critical care compared with those who were unexposed, as shown in Figure 2A. Specifically, 58.9% of exposed patients experienced the composite adverse outcome compared with 26.8% of unexposed patients; patients prescribed benzodiazepines, antipsychotics, or Alzheimer’s disease medications were 2.20 times as likely to experience the composite adverse outcome as those who were unexposed (RR = 2.20; 95% CI, 1.35–3.57). In absolute terms, the exposed group had a 32.1% higher risk of the outcome compared with the unexposed group (risk difference = 0.32; 95% CI, 0.15–0.50; *p* = 0.001). Additionally, the odds of the outcome were 3.92 times higher in the exposed group (odds ratio = 3.92; 95% CI, 1.77–8.70).

### PHM exposure and time to HBAC services

After matching, there was a significant difference in the time to event between the exposed and unexposed groups, with only 12.8% of patients in the exposed group remaining event-free, compared with 62.7% in the unexposed group. The median survival time was reported only for the exposed group (201 days). This difference in survival between groups was statistically significant (log-rank test: *χ*^2^ = 10.965, *p* = 0.0009). Furthermore, the PHM-exposed group had a 2.69-fold higher hazard of experiencing the event compared with the unexposed group throughout the follow-up [Figure 2B; hazard ratio (HR) = 2.69; 95% CI, 1.46–4.98].

## Discussion

In this study, we aimed to assess prescribing patterns following an FTD diagnosis and to evaluate the association between exposure to PHMs and adverse outcomes. We identified an elevated risk of hospitalization, an emergency department visit, or critical care for individuals who were prescribed benzodiazepines, Alzheimer’s disease medications, or antipsychotic medications following diagnosis, when compared with those who were unexposed. Our findings describe real-world prescribing in FTD and its association with acute-care use.

When assessing pharmacological treatment patterns following a diagnosis of FTD, we found that more than half of the cohort (61.5%) was prescribed no medications included in the study exposure definition: Alzheimer’s disease medications, antipsychotics, SSRIs, trazodone, and benzodiazepines. As there are currently no disease-modifying medications available for FTD treatment, this conservative strategy may be adequate depending on the patient’s clinical syndrome, as evaluated by the physician (18). However, benzodiazepines emerged as the most frequently prescribed drug class among the queried pharmacological exposures and the most common pharmacological intervention following an EHR-recorded diagnosis of FTD in this cohort, followed by the prescription of antipsychotics and Alzheimer’s disease medications; their association with falls, pneumonia, hospitalization, and mortality in older adults is well documented (19–22). Our findings are consistent with that class effect and may partly reflect it. Whether FTD confers additional vulnerability is a separate question our design cannot answer, as we had no comparison cohort of older adults without FTD; thus, the estimate is internal to the disease population. Comparing benzodiazepine-exposed patients with and without FTD, matched for age and comorbidity, would help address this question. Moreover, despite clinical guidelines advising against their use for neuropsychiatric symptoms in dementia (23), our data suggest that they remain a common choice in FTD patients, which may be due to the management challenges of behavioral symptoms or a lack of familiarity with safer alternatives (24). That said, prescription of trazodone and SSRIs was infrequent in this cohort, limiting comparative interpretation.

Although both exposed and unexposed groups were at risk for adverse outcomes, exposure to medications potentially harmful in FTD—benzodiazepines, typical antipsychotics, and Alzheimer’s disease drugs—was associated with a 2.20-fold higher risk of emergency department visits, inpatient hospitalizations, and critical care needs during follow-up. Accordingly, a greater proportion of exposed patients experienced HBAC, and the time to the first event was shorter, suggesting that PHM exposure may identify a higher-risk subgroup.

This study has several strengths. To our knowledge, it is the first to leverage the TriNetX database to investigate FTD, a condition that poses unique research challenges due to the typically small patient numbers observed in non-specialist centers. Furthermore, it represents the first real-world analysis of treatment patterns and prescription practices for FTD following diagnosis. The dynamic nature of the TriNetX platform, which continuously updates patient information, ensures that the data analyzed are current and reflective of evolving clinical practice. In addition, recent evidence has highlighted TriNetX as a valuable platform for real-world evidence, particularly in the context of randomized controlled trials involving off-label medications and rare diseases, further underscoring its suitability for studying conditions such as FTD (25). Our eligibility criteria reinforced this: Excluding any patient carrying a code from the F01–F09 block, which includes unspecified dementia and delirium, removed 6,989 of the 7,242 patients initially identified and accounted for almost all of the attrition. We applied this exclusion to prevent two forms of contamination—coexisting Alzheimer’s disease or unspecified dementia carries its own prescribing indications, and delirium codes mark both a reason for sedative prescribing and a driver of acute care—so the retained cohort carries a specific FTD code and no competing dementia or delirium code, which strengthens the exposure–outcome comparison.

Nonetheless, some limitations warrant consideration. First, the exposed and unexposed cohorts in Compare Outcomes were not anchored to the same index date. The unexposed cohort’s index date was the diagnosis date. The exposed cohort’s index date was tied to both the diagnosis and the qualifying prescription, since receiving that prescription within 1 year of diagnosis was part of how the exposed cohort was defined. In practice, this means the exposed cohort’s index date may fall later than diagnosis – at or after the first prescription – rather than at diagnosis for both groups equally. This design introduces a period of immortal-time bias—the time between diagnosis and prescription is not counted as at-risk time for exposed patients. A separate concern is ascertainment: Every exposed patient had at least one documented health-system contact (their prescription) during the qualifying window; therefore, some of the observed difference could reflect closer monitoring rather than true risk. Fully correcting both issues would require patient-level data and time-varying exposure modeling, which this platform does not provide. Relatedly, 15 exposed patients had a qualifying HBAC event at or before their index date. Because the exposed cohort’s index date is tied to the qualifying prescription, these acute events in some cases may have preceded or possibly prompted the prescription rather than the reverse. This is distinct from, but compounds, the immortal-time concern above.

Second, death was not treated as a competing event. TriNetX censors each patient after the last recorded event in their record, and the platform does not distinguish death from disenrollment or transfer of care outside the network; therefore, we cannot confirm that censoring occurred specifically at death. One consideration limits, without removing, this concern: A majority of deaths occurring within the health system would have followed an inpatient, emergency department, or critical care encounter, so those patients would already have contributed to the composite outcome before dying.

Third, the dataset does not provide information on the temporal order of symptom onset, comprehensive psychiatric assessments, imaging reports, or the specific clinical rationale behind each prescription, and TriNetX does not reliably distinguish bvFTD from PPA variants, limiting adjustment for phenotype-specific neuropsychiatric burden and prescribing patterns.

Fourth, exposure was defined based on prescription orders, and TriNetX does not capture pharmacy dispensing or fill data; therefore, we cannot confirm that prescribed medications were actually obtained or taken. Medication non-adherence is common in dementia and may be more pronounced with cognitive impairment; such non-differential exposure misclassification would generally bias associations toward the null; therefore, the true effect of PHM use, when actually taken, could be larger than what we observed.

Fifth, the diagnostic trajectories of patients—whether sporadic or genetically determined—were not accessible, and it remains unclear whether the individuals included were evaluated at specialized centers or in general clinics. It is plausible that patients who engaged with multiple healthcare providers prior to receiving a diagnosis of bvFTD were more likely to receive additional psychotropic medications, which could affect the overall results. Relatedly, C9orf72 repeat expansion causes approximately 25% of familial and 6% of sporadic FTD cases in populations of European ancestry (26) and produces prominent psychotic symptoms in many carriers. For such patients, an antipsychotic prescription may be clinically appropriate, and our exposure definition cannot make that distinction because genetic status was also unavailable. This applies to a minority of the cohort, but it qualifies any reading of antipsychotic exposure as harmful in every case.

Sixth, the analytical framework within TriNetX imposes a constraint on data granularity: Patient counts greater than zero but fewer than 10 in a given category are automatically rounded up to 10 to safeguard anonymity. This masking procedure was primarily observed in certain demographic subgroups and medication categories, potentially introducing imbalance in propensity score matching and bias in analyses of infrequent exposures. This constraint also made class-specific analyses infeasible: stratifying the 71 exposed patients across three drug classes and then by outcome placed most cells below the reporting threshold, and the platform returned no result. The same constraint prevented a sensitivity analysis excluding midazolam, which matters because midazolam is often given for procedural or emergency sedation rather than for longitudinal symptom management, and such prescriptions may follow acute care contact rather than precede it. Similarly, platform constraints prevented a sensitivity analysis restricted to patients with multiple FTD diagnosis codes or a specialist encounter, which would strengthen diagnostic certainty and should be prioritized in future replication studies. Supplementary Table 3 reports patient counts for every drug in the query.

It is also important to state that TriNetX data come mostly from insured patients in academic and acute care settings in the United States. This limitation narrows the population further: Patients with mixed, uncertain, or evolving diagnoses, common in routine dementia care, may be underrepresented, and the retained cohort is likely enriched for patients observed in specialist settings, so our estimates describe patients with a clean, specific FTD record rather than the wider population managed as FTD in practice. In addition, TriNetX updates its records continuously; thus, the cohort may differ between query dates, which may limit exact reproducibility of the analysis. However, this limitation is inherent to the TriNetX environment and has been acknowledged in previous studies using the same platform, highlighting the need for cautious interpretation (27).

Seventh, patients prescribed benzodiazepines or antipsychotics likely represent individuals with more severe neuropsychiatric symptoms, behavioral dysregulation, or caregiver burden—all factors that may independently increase HBAC utilization regardless of medication exposure. Although propensity-score matching was performed to balance demographic and clinical variables between groups and reduce confounding, residual confounding related to disease severity and neuropsychiatric burden could not be fully accounted for using the available dataset. Therefore, these findings should not be interpreted as definitive evidence of medication-induced harm. Given this limitation and the temporal misclassification described above, we frame the exposure–outcome analysis as exploratory rather than confirmatory: It identifies an association worth further study, but not a causal estimate of harm.

Despite these limitations, the use of a large, real-world, multi-institutional dataset allows for a broad characterization of treatment practices across diverse clinical settings, contributing valuable insights into the management of FTD across routine clinical care. Our findings indicate that most treated patients received drugs with weak efficacy evidence and known safety concerns in dementia and that this exposure marked a subgroup in which a higher proportion experienced HBAC and the time to the first event was shorter. Whether changes in prescribing improve outcomes requires prospective study.

## Data Availability

The data analyzed in this study come from the TriNetX Research Network and are not publicly available. TriNetX is a proprietary federated network, and access is licensed to investigators at member institutions. The authors held such a licence, which has since expired. Aggregate results supporting the findings, including the query specification (Supplementary Tables 1, 4, 5), diagnostic and procedural code lists (Supplementary Tables 4, 5), and cohort attrition counts (Supplementary Table 7), appear in the Supplementary material. Requests for access to the underlying data should be directed to TriNetX LLC (https://trinetx.com).
