# Supplementary material for: Pharmacological management in frontotemporal dementia and patient outcomes: a retrospective cohort study using the TriNetX platform
Source: Front Neurol. 2026 Sep 3;17:1930046. doi: 10.3389/fneur.2026.1930046 (PMC13583036; doi:10.3389/fneur.2026.1930046)
Supplement: Supplementary file 1 [file Data_Sheet_1.DOCX]

**SUPPLEMENTARY MATERIAL**

**E1. TriNetX Data Source**

**eTable 1 – Summary Statistics**

**eTable1 – Query Criteria for Lines of Treatment**

**eTable 2 – Treatment Definitions**

**eTable 3 – Specific Prescriptions**

**eTable 4 – Unexposed Group Cohort Criteria**

**eTable 5 – Exposed Group Cohort Criteria**

**eTable 6 – Outcome Definitions**

**E2. Statistical Analysis**

**eTable 7 – Propensity Score Matching**

**eTable 8 – Matching Criteria**

**eTable 9 – Follow-up Time**

**E1. TriNetX Data Source**

TriNetX is the global federated health research network providing access to electronic medical records (diagnoses, procedures, medications, laboratory values, genomic information) across large healthcare organizations (HCOs).

The available data included information about the demographics, diagnoses (based on the International Classification of Diseases, Tenth Revision, Clinical Modification, ICD-10-CM codes), procedures (coded in The International Classification of Diseases, Tenth Revision, Procedure Coding System, ICD-10-PCS or Current Procedural Terminology, CPT), medication (coded in Veterans Affairs National Formulary), laboratory tests (coded in Logical Observation Identifiers Names and Codes, LOINC), genomics (coded in Human Genome Variation Society, HGVS), and healthcare utilization. TriNetX does not allow data downloads, or individual patient data for review. However, it allows analysis in the form of queries.

In order to guarantee patient anonymity, TriNetX sets a virtual value of 10 every time patient counts fall below this value in a category. This descriptive characteristic does not interfere with data analysis in the platform.

**eFigure 1 – Summary Statistics**

**eFigure 1** **- Demographics of study participants**: Figure presents the data collected from participating patients and the data available on the platform, as well as the participants' places of origin.

**E1.1 Lines of Treatment Cohort**

Analysis process in Line of Treatment includes two main steps: 1) Define the population for the analysis through setting query criteria; 2) Set up analysis, select treatments, criteria for lines of treatment and the time frame, run analysis on the selected treatments.

**eTable1 – Query Criteria for Lines of Treatment**

We used this query scheme because it matches current diagnostic criteria for probable frontotemporal dementia, which is considered to patients who present at least three of the six main criteria, characteristic of FTD, with a perception of worsening of the symptoms through time. These changes are: early disinhibition; early apathy or inertia; early loss of empathy/sympathy; early perseverative, stereotyped, or compulsive/ritualistic behavior; hyperorality and eating disorders; and a neuropsychological profile with executive dysfunction and relative preservation of episodic memory and visuospatial abilities. The FTD diagnosis becomes probable when the patient starts presenting a dependence in its day-to-day activities. These clinical characteristics are seen by the clinician. The diagnosis, to be probable, also requires the presence of a neuroimaging exam that shows atrophy in frontal and temporal lobes. Besides, we aimed to evaluate current practices, hence the five-year time restraint.

| Ungrouped terms | | | | | |
| --- | --- | --- | --- | --- | --- |
|  | cannot have |  | diagnosis | UMLS:ICD10CM:F01-F09 | Mental disorders due to known physiological conditions |
|  |  | or | diagnosis | UMLS:ICD10CM:G31.83 | Neurocognitive disorder with Lewy bodies |
|  |  | or | diagnosis | UMLS:ICD10CM:G30 | Alzheimer's disease |
|  |  | or | diagnosis | UMLS:ICD10CM:G32 | Other degenerative disorders of nervous system in diseases classified elsewhere |
|  |  | or | diagnosis | UMLS:ICD10CM:G31.1 | Senile degeneration of brain, not elsewhere classified |
|  |  | or | diagnosis | UMLS:ICD10CM:G31.2 | Degeneration of nervous system due to alcohol |
|  |  | or | diagnosis | UMLS:ICD10CM:G31.8 | Other specified degenerative diseases of nervous system |
|  |  | or | diagnosis | UMLS:ICD10CM:G31.9 | Degenerative disease of nervous system, unspecified |
|  |  | or | diagnosis | UMLS:ICD9CM:330 | Cerebral degenerations usually manifest in childhood |
|  |  | or | diagnosis | UMLS:ICD9CM:331 | Other cerebral degenerations |
| Group 1 | | | | | |
|  | **Group 1A Diagnosis** | | | | |
|  | must have |  | diagnosis | UMLS:ICD10CM:G31.0 | Frontotemporal dementia |
|  | date constraint | | This group occurred since 5 years ago | | |
|  | event relationship | | Any instance of Outpatient visit occurred within 1 year on or after any instance of Diagnosis | | |
|  | **Group 1B Outpatient visit** | | | | |
|  | must have | any of | procedure | UMLS:CPT:1013626 | Office or Other Outpatient Services |
|  |  |  | procedure | UMLS:CPT:1013687 | Office or Other Outpatient Consultations |
|  |  |  | visit | UMLS:HL7V3.0:VisitType:AMB | Visit: Ambulatory |
| Group 2 | | | | | |
|  | **Group 2A Diagnosis** | | | | |
|  | must have |  | diagnosis | UMLS:ICD10CM:G31.0 | Frontotemporal dementia |
|  | date constraint | | This group occurred since 5 years ago | | |
|  | event relationship | | Any instance of Exam occurred on or after any instance of Diagnosis | | |
|  | **Group 2B Exam** | | | | |
|  | must have | any of | procedure | UMLS:CPT:1010296 | Computed tomography, head or brain |
|  |  |  | procedure | UMLS:CPT:1010326 | Magnetic resonance (eg, proton) imaging, brain (including brain stem) |

**eTable 1** **– Query Criteria for Lines of Treatment Analysis**: Patient inclusion criteria and event grouping for frontotemporal dementia (FTD) cohorts. Ungrouped terms exclude patients with other neurodegenerative or secondary dementias (e.g., Alzheimer’s disease, Lewy body dementia, alcohol-related or unspecified degenerative brain disorders). Group 1 includes patients with a FTD diagnosis within the last 5 years and an outpatient visit within 1 year of diagnosis. Group 2 includes patients with a FTD diagnosis in the last 5 years and a brain imaging exam (CT or MRI) on or after diagnosis. Standardized codes are provided using ICD-9/ICD-10 for diagnoses and CPT/HL7 for procedures and visit types.

**eTable 2 – Treatment Definitions**

We included the specified medications in our study because they reflect current clinical practices and the ongoing debate regarding pharmacological management in frontotemporal dementia (FTD). Benzodiazepines and antipsychotics, although commonly used to control agitation and inappropriate behaviors, are considered potentially inappropriate medications (PIMs) in FTD, as their effects are not validated by clinical trials and consensus guidelines generally advise against their use. Similarly, anticholinesterases and memantine, standard treatments for Alzheimer’s disease, have been shown to be ineffective or even detrimental in FTD, such as worsening agitation. On the other hand, Selective Serotonin Reuptake Inhibitors (SSRIs), particularly trazodone, have the strongest evidence for symptom management, while new treatments like intranasal oxytocin show promise in clinical trials. Evaluating the use of these medications allows us to assess real-world prescribing patterns, adherence to guidelines, and the prevalence of both evidence-based and potentially inappropriate treatments in FTD management.

| Benzodiazepines | | | | |
| --- | --- | --- | --- | --- |
|  | **Treatment definition** | | | |
|  | | Medication | NLM:RXNORM:2598 | clonazepam |
|  | | Medication | NLM:RXNORM:6470 | lorazepam |
|  | | Medication | NLM:RXNORM:6960 | midazolam |
|  | | Medication | NLM:RXNORM:596 | alprazolam |
|  | | Medication | NLM:RXNORM:3322 | diazepam |
| Antipsychotics | | | | |
|  | **Treatment definition** | | | |
|  | | Medication | NLM:RXNORM:2626 | clozapine |
|  | | Medication | NLM:RXNORM:35636 | risperidone |
|  | | Medication | NLM:RXNORM:89013 | aripiprazole |
|  | | Medication | NLM:RXNORM:35636 | risperidone |
|  | | Medication | NLM:RXNORM:51272 | quetiapine |
|  | | Medication | NLM:RXNORM:61381 | olanzapine |
|  | | Medication | NLM:RXNORM:5093 | haloperidol |
|  | | Medication | NLM:RXNORM:2403 | chlorpromazine |
| SSRIs | | | | |
|  | **Treatment definition** | | | |
|  | | Medication | NLM:RXNORM:2556 | citalopram |
|  | | Medication | NLM:RXNORM:32937 | paroxetine |
|  | | Medication | NLM:RXNORM:42355 | fluvoxamine |
| Adequate for FTD treatment | | | | |
|  | **Treatment definition** | | | |
|  | | Medication | NLM:RXNORM:10737 | trazodone |
|  | | Medication | NLM:RXNORM:7824 | oxytocin |
| Alzheimer’s Disease Medications | | | | |
|  | **Treatment definition** | | | |
|  | | Medication | NLM:RXNORM:6719 | memantine |
|  | | Medication | NLM:RXNORM:135447 | donepezil |
|  | | Medication | NLM:RXNORM:4637 | galantamine |
|  | | Medication | NLM:RXNORM:183379 | rivastigmine |

**eTable 2 -** **Treatment Definitions for Lines of Treatment Analysis**: Medications included in the study, grouped by pharmacological class and relevance to frontotemporal dementia (FTD) treatment. NLM:RXNORM codes provide standardized drug identifiers from the U.S. National Library of Medicine for consistent medication identification across studies.

**eTable 3 – Specific Prescriptions**

| Treatment | Patients |
| --- | --- |
| Lorazepam | 17 |
| Midazolam | 11 |
| Donepezil | 3 |
| Olanzapine | 3 |
| Alprazolam | 2 |
| Haloperidol \| midazolam | 1 |
| Diazepam | 2 |
| Lorazepam \| midazolam | 2 |
| Midazolam \| alprazolam | 1 |
| Memantine | 1 |
| Memantine \| donepezil | 1 |
| Galantamine | 1 |
| Rivastigmine | 1 |
| Fluvoxamine | 1 |
| Trazodone | 1 |
| Citalopram \| trazodone | 0 |
| Oxytocin | 1 |
| Aripiprazole | 1 |
| Trazodone \| aripiprazole | 1 |
| Risperidone \| risperidone | 1 |
| Quetiapine | 1 |
| Donepezil \| quetiapine | 1 |
| Donepezil \| olanzapine | 1 |
| Citalopram \| olanzapine | 0 |
| Quetiapine \| olanzapine | 1 |
| Haloperidol | 1 |
| Clonazepam | 0 |
| Olanzapine \| haloperidol \| clonazepam | 1 |
| Fluvoxamine \| lorazepam | 0 |
| Trazodone \| lorazepam | 0 |
| Paroxetine \| aripiprazole \| lorazepam | 0 |
| Risperidone \| risperidone \| lorazepam | 1 |
| Quetiapine \| lorazepam | 1 |
| Olanzapine \| lorazepam | 0 |
| Haloperidol \| lorazepam | 1 |
| Quetiapine \| haloperidol \| lorazepam | 1 |
| Risperidone \| risperidone \| olanzapine \| haloperidol \| lorazepam | 0 |
| Trazodone \| midazolam | 0 |
| Quetiapine \| lorazepam \| midazolam | 1 |
| Citalopram \| haloperidol \| lorazepam \| midazolam | 1 |
| Memantine \| alprazolam | 1 |
| Donepezil \| alprazolam | 0 |
| Trazodone \| alprazolam | 1 |
| Citalopram \| trazodone \| alprazolam | 0 |
| Olanzapine \| lorazepam \| alprazolam | 1 |
| Memantine \| donepezil \| diazepam | 1 |
| Quetiapine \| olanzapine \| lorazepam \| diazepam | 1 |

**eTable 3 – Specific Prescriptions:** Values represent the total count of patients treated with each specific medication included in the study.

**E1.2 Compare Outcomes Cohort**

In Compare Outcomes, the analysis process includes two main steps: 1) Defining the cohorts through query criteria; 2) Setting up and running the analysis. Setting up the analysis requires definitions for the index event, outcomes criteria, and the time frame. Compare outcomes supports four analyses: Measures of Association, Survival, Number of Instances and Lab result distribution.

**eTable 4 – Unexposed Group Cohort Criteria**

| Ungrouped terms | | | | | |
| --- | --- | --- | --- | --- | --- |
|  | cannot have |  | diagnosis | UMLS:ICD10CM:F01-F09 | Mental disorders due to known physiological conditions |
|  |  | or | diagnosis | UMLS:ICD10CM:G31.83 | Neurocognitive disorder with Lewy bodies |
|  |  | or | diagnosis | UMLS:ICD10CM:G30 | Alzheimer's disease |
|  |  | or | diagnosis | UMLS:ICD10CM:G32 | Other degenerative disorders of nervous system in diseases classified elsewhere |
|  |  | or | diagnosis | UMLS:ICD10CM:G31.1 | Senile degeneration of brain, not elsewhere classified |
|  |  | or | diagnosis | UMLS:ICD10CM:G31.2 | Degeneration of nervous system due to alcohol |
|  |  | or | diagnosis | UMLS:ICD10CM:G31.8 | Other specified degenerative diseases of nervous system |
|  |  | or | diagnosis | UMLS:ICD10CM:G31.9 | Degenerative disease of nervous system, unspecified |
|  |  | or | diagnosis | UMLS:ICD9CM:330 | Cerebral degenerations usually manifest in childhood |
|  |  | or | diagnosis | UMLS:ICD9CM:331 | Other cerebral degenerations |
| Group 1 | | | | | |
|  | **Group 1A Diagnosis** | | | | |
|  | must have |  | diagnosis | UMLS:ICD10CM:G31.0 | Frontotemporal dementia |
|  | date constraint | | This group occurred since 5 years ago | | |
|  | event relationship | | Any instance of Outpatient visit occurred within 1 year on or after any instance of Diagnosis | | |
|  | **Group 1B Outpatient visit** | | | | |
|  | must have | any of | procedure | UMLS:CPT:1013626 | Office or Other Outpatient Services |
|  |  |  | procedure | UMLS:CPT:1013687 | Office or Other Outpatient Consultations |
|  |  |  | visit | UMLS:HL7V3.0:VisitType:AMB | Visit: Ambulatory |
| Group 2 | | | | | |
|  | **Group 2A Diagnosis** | | | | |
|  | must have |  | diagnosis | UMLS:ICD10CM:G31.0 | Frontotemporal dementia |
|  | date constraint | | This group occurred since 5 years ago | | |
|  | event relationship | | Any instance of Exam occurred on or after any instance of Diagnosis | | |
|  | **Group 2B Exam** | | | | |
|  | must have | any of | procedure | UMLS:CPT:1010296 | Computed tomography, head or brain |
|  |  |  | procedure | UMLS:CPT:1010326 | Magnetic resonance (eg, proton) imaging, brain (including brain stem) |
| Group 3 | | | | | |
|  | **Group 3A DX** | | | | |
|  | must have |  | diagnosis | UMLS:ICD10CM:G31.0 | Frontotemporal dementia |
|  |  | and any of | procedure | UMLS:CPT:1013626 | Office or Other Outpatient Services |
|  |  |  | procedure | UMLS:CPT:1013687 | Office or Other Outpatient Consultations |
|  |  |  | visit | UMLS:HL7V3.0:VisitType:AMB | Visit: Ambulatory |
|  | date constraint | | This group occurred since 5 years ago | | |
|  | event relationship | | Any instance of Treatment occurred within 1 year on or after any instance of DX | | |
|  | **Group 3B Treatment** | | | | |
|  | cannot have |  | medication | NLM:RXNORM:3322 | diazepam |
|  |  | or | medication | NLM:RXNORM:2598 | clonazepam |
|  |  | or | medication | NLM:RXNORM:6960 | midazolam |
|  |  | or | medication | NLM:RXNORM:596 | alprazolam |
|  |  | or | medication | NLM:RXNORM:6470 | lorazepam |
|  |  | or | medication | NLM:RXNORM:2626 | clozapine |
|  |  | or | medication | NLM:RXNORM:61381 | olanzapine |
|  |  | or | medication | NLM:RXNORM:51272 | quetiapine |
|  |  | or | medication | NLM:RXNORM:35636 | risperidone |
|  |  | or | medication | NLM:RXNORM:89013 | aripiprazole |
|  |  | or | medication | NLM:RXNORM:5093 | haloperidol |
|  |  | or | medication | NLM:RXNORM:2403 | chlorpromazine |
|  |  | or | medication | NLM:RXNORM:135447 | donepezil |
|  |  | or | medication | NLM:RXNORM:4637 | galantamine |
|  |  | or | medication | NLM:RXNORM:6719 | memantine |
|  |  | or | medication | NLM:RXNORM:183379 | rivastigmine |

**eTable 4 – Unexposed Group Cohort Criteria:** “Ungrouped terms” lists diagnoses excluded from all groups. Groups 1–3 specify required diagnoses, procedures, or visits, along with temporal relationships between events. Group 3B additionally lists medications that patients must not have received. All entries are coded using standard terminologies (ICD-10-CM, ICD-9-CM, CPT, HL7, RxNorm).

**eTable 5 – Exposed Group Cohort Criteria**

| Ungrouped terms | | | | | |
| --- | --- | --- | --- | --- | --- |
|  | cannot have |  | diagnosis | UMLS:ICD10CM:F01-F09 | Mental disorders due to known physiological conditions |
|  |  | or | diagnosis | UMLS:ICD10CM:G31.83 | Neurocognitive disorder with Lewy bodies |
|  |  | or | diagnosis | UMLS:ICD10CM:G30 | Alzheimer's disease |
|  |  | or | diagnosis | UMLS:ICD10CM:G32 | Other degenerative disorders of nervous system in diseases classified elsewhere |
|  |  | or | diagnosis | UMLS:ICD10CM:G31.1 | Senile degeneration of brain, not elsewhere classified |
|  |  | or | diagnosis | UMLS:ICD10CM:G31.2 | Degeneration of nervous system due to alcohol |
|  |  | or | diagnosis | UMLS:ICD10CM:G31.8 | Other specified degenerative diseases of nervous system |
|  |  | or | diagnosis | UMLS:ICD10CM:G31.9 | Degenerative disease of nervous system, unspecified |
|  |  | or | diagnosis | UMLS:ICD9CM:330 | Cerebral degenerations usually manifest in childhood |
|  |  | or | diagnosis | UMLS:ICD9CM:331 | Other cerebral degenerations |
| Group 1 | | | | | |
|  | **Group 1A Diagnosis** | | | | |
|  | must have |  | diagnosis | UMLS:ICD10CM:G31.0 | Frontotemporal dementia |
|  | date constraint | | This group occurred since 5 years ago | | |
|  | event relationship | | Any instance of Outpatient visit occurred within 1 year on or after any instance of Diagnosis | | |
|  | **Group 1B Outpatient visit** | | | | |
|  | must have | any of | procedure | UMLS:CPT:1013626 | Office or Other Outpatient Services |
|  |  |  | procedure | UMLS:CPT:1013687 | Office or Other Outpatient Consultations |
|  |  |  | visit | UMLS:HL7V3.0:VisitType:AMB | Visit: Ambulatory |
| Group 2 | | | | | |
|  | **Group 2A Diagnosis** | | | | |
|  | must have |  | diagnosis | UMLS:ICD10CM:G31.0 | Frontotemporal dementia |
|  | date constraint | | This group occurred since 5 years ago | | |
|  | event relationship | | Any instance of Exam occurred on or after any instance of Diagnosis | | |
|  | **Group 2B Exam** | | | | |
|  | must have | any of | procedure | UMLS:CPT:1010296 | Computed tomography, head or brain |
|  |  |  | procedure | UMLS:CPT:1010326 | Magnetic resonance (eg, proton) imaging, brain (including brain stem) |
| Group 3 | | | | | |
|  | **Group 3A DX** | | | | |
|  | must have |  | diagnosis | UMLS:ICD10CM:G31.0 | Frontotemporal dementia |
|  |  | and any of | procedure | UMLS:CPT:1013626 | Office or Other Outpatient Services |
|  |  |  | procedure | UMLS:CPT:1013687 | Office or Other Outpatient Consultations |
|  |  |  | visit | UMLS:HL7V3.0:VisitType:AMB | Visit: Ambulatory |
|  | date constraint | | This group occurred since 5 years ago | | |
|  | event relationship | | Any instance of Treatment occurred within 1 year on or after any instance of DX | | |
|  | **Group 3B Treatment** | | | | |
|  | must have | any of | medication | NLM:RXNORM:3322 | diazepam |
|  |  |  | medication | NLM:RXNORM:2598 | clonazepam |
|  |  |  | medication | NLM:RXNORM:6960 | midazolam |
|  |  |  | medication | NLM:RXNORM:596 | alprazolam |
|  |  |  | medication | NLM:RXNORM:61381 | olanzapine |
|  |  |  | medication | NLM:RXNORM:6470 | lorazepam |
|  |  |  | medication | NLM:RXNORM:2626 | clozapine |
|  |  |  | medication | NLM:RXNORM:51272 | quetiapine |
|  |  |  | medication | NLM:RXNORM:89013 | aripiprazole |
|  |  |  | medication | NLM:RXNORM:2403 | chlorpromazine |
|  |  |  | medication | NLM:RXNORM:5093 | haloperidol |
|  |  |  | medication | NLM:RXNORM:183379 | rivastigmine |
|  |  |  | medication | NLM:RXNORM:135447 | donepezil |
|  |  |  | medication | NLM:RXNORM:6719 | memantine |
|  |  |  | medication | NLM:RXNORM:4637 | galantamine |
|  |  |  | medication | NLM:RXNORM:35636 | risperidone |

**eTable 5 – Exposed Group Cohort Criteria:** “Ungrouped terms” lists diagnoses excluded from all groups. Groups 1–3 specify required diagnoses, procedures, or visits, along with temporal relationships between events. Group 3B additionally lists medications that patients must have received. All entries are coded using standard terminologies (ICD-10-CM, ICD-9-CM, CPT, HL7, RxNorm).

**eTable 6 – Hospital Based Acute Care Outcome Definitions**

| HBAC Outcome | | | | |
| --- | --- | --- | --- | --- |
|  | **Outcome definition** | | | |
|  | | Procedure | UMLS:CPT:1013659 | Hospital Inpatient and Observation Care Services |
|  | | Procedure | UMLS:CPT:1013711 | Emergency Department Services |
|  | | Procedure | UMLS:CPT:1013729 | Critical Care Services |
|  | **Settings for the performed analyses** | | | |
|  | | Kaplan - Meier survival analysis | | including patients with outcome prior to the time window |
|  | | Risk analysis | | including patients with outcome prior to the time window |
|  | | Number of instances analysis | | including patients with outcome prior to the time window excluding patients with zero outcomes counts are grouped by date |

**eTable 6 – Hospital Based Acute Care Outcome Definitions:** Outcomes were defined based on procedures: hospital inpatient and observation care (UMLS:CPT:1013659), emergency department services (UMLS:CPT:1013711), and critical care services (UMLS:CPT:1013729). Analyses included Kaplan–Meier survival, risk, and number-of-instances approaches, all incorporating patients with prior outcomes and excluding those with zero events. Counts are aggregated by date.

**E2. Statistical Analysis**

**eTable 7 – Propensity Score Matching**

| **Cohort 1 and cohort 2 patient count before and after propensity score matching** | | | | | |
| --- | --- | --- | --- | --- | --- |
|  | | Cohort | Patient count before matching | | Patient count after matching |
|  | | 1 - Not exposed | 108 | | 56 |
|  | | 2 - Exposed | 71 | | 56 |
| **Propensity score density function - Before and after matching (cohort 1 - purple, cohort 2 - green)** | | | | | |
|  |  |  | |  | |

**eTable 7 – Propensity Score Matching:** Patient counts in Cohorts 1 (Not exposed) and 2 (Exposed) before and after propensity score matching. The table shows total patients prior to matching and the matched cohort size. Propensity score density functions are shown for each cohort (Cohort 1: purple; Cohort 2: green) before and after matching, illustrating improved balance between groups.

**eTable 8 – Matching Criteria**

| **Diagnosis** | | | | | | | | |
| --- | --- | --- | --- | --- | --- | --- | --- | --- |
|  | Cohort | |  | Mean ± SD | Patients | % of Cohort | P-Value | Std diff. |
|  | 1 2 | Z00-Z99 | Factors influencing health status and contact with health services |  | 38 37 | 67.9% 66.1% | 0.841 | 0.038 |
|  | 1 2 | I00-I99 | Diseases of the circulatory system |  | 35 36 | 62.5% 64.3% | 0.844 | 0.037 |
|  | 1 2 | E00-E89 | Endocrine, nutritional and metabolic diseases |  | 41 40 | 73.2% 71.4% | 0.833 | 0.040 |
|  | 1 2 | C00-D49 | Neoplasms |  | 17 18 | 30.4% 32.1% | 0.838 | 0.039 |
|  | 1 2 | F01-F99 | Mental, Behavioral and Neurodevelopmental disorders |  | 27 27 | 48.2% 48.2% | 1 | <0.001 |
|  | 1 2 | V00-Y99 | External causes of morbidity |  | 18 17 | 32.1% 30.4% | 0.838 | 0.039 |
| **Procedure** | | | | | | | | |
|  | Cohort | |  | Mean ± SD | Patients | % of Cohort | P-Value | Std diff. |
|  | 1 2 | 1013659 | Hospital Inpatient and Observation Care Services |  | 21 21 | 37.5% 37.5% | 1 | <0.001 |
|  | 1 2 | 1013711 | Emergency Department Services |  | 27 24 | 48.2% 42.9% | 0.569 | 0.108 |
|  | 1 2 | 1013729 | Critical Care Services |  | 10 13 | 17.9% 23.2% | 0.483 | 0.133 |
| **Laboratory** | | | | | | | | |
|  | Cohort | |  | Mean ± SD | Patients | % of Cohort | P-Value | Std diff. |
|  | 1 2 | 9083 | BMI | 25.3 +/- 6.5 27.2 +/- 4.4 | 33 35 | 58.9% 62.5% | 0.171 | 0.334 |
|  | 1 2 |  | 0 - 0 kg/m2 |  | 33 35 | 58.9% 62.5% | 0.699 | 0.073 |

**eTable 8 – Matching Criteria:** Baseline characteristics of cohorts used for matching are presented. Cohort 1 = Not exposed; Cohort 2 = Exposed. Continuous variables are shown as mean ± SD. “Patients” indicates the number with the characteristic; “% of Cohort” shows its proportion. P-values <0.05 indicate significant differences; standardized differences <0.1 indicate adequate balance. Diagnoses (ICD-10), procedures (CPT/HCPCS), and BMI (9083) are included. This table evaluates cohort comparability before and after matching.

**eTable 9 – Follow-up Time**

| **Follow-up Time (Before Matching)** | | | | | | |
| --- | --- | --- | --- | --- | --- | --- |
|  |  | Cohort | Mean Follow-up (Days) | Standard Deviation | Median Follow-up (Days) | Interquartile Range |
|  |  | Not exposed | 511.130 | 506.496 | 302 | 814 |
|  |  | Exposed | 409.592 | 393.965 | 309 | 475 |
|  | | | | | | |
|  | | | | | | |
| **Follow-up Time (After Matching)** | | | | | | |
|  |  | Cohort | Mean Follow-up (Days) | Standard Deviation | Median Follow-up (Days) | Interquartile Range |
|  |  | Not exposed | 520.375 | 480.706 | 374 | 774.500 |
|  |  | Exposed | 435.286 | 393.603 | 321.500 | 548.500 |
|  | | | | | | |

**eTable 9 – Follow-up Time:** Table shows follow-up duration (days) for Not exposed and Exposed cohorts before and after matching. Data include mean ± standard deviation, median, and interquartile range (IQR).
